# Optimal Energy-Delay in Energy Harvesting Wireless Sensor Networks with Interference Channels

**DOI:** 10.3390/s19040785

**Published:** 2019-02-14

**Authors:** Dongbin Jiao, Liangjun Ke, Shengbo Liu, Felix T.S. Chan

**Affiliations:** 1 State Key Laboratory for Manufacturing Systems Engineering, Xi’an Jiaotong University, Xi’an 710049, China; 2 School of Electronic and Information Engineering, Xi’an Jiaotong University, Xi’an 710049, China; 3 School of Information Science and Engineering, Xiamen University, Xiamen 361005, China; liushb@stu.xmu.edu.cn; 4 Key Laboratory of Underwater Acoustic Communication and Marine Information Technology Ministry of Education, Xiamen University, Xiamen 361005, China; 5 Department of Industrial and Systems Engineering, The Hong Kong Polytechnic University, Hung Hom, Hong Kong, China; f.chan@polyu.edu.hk

**Keywords:** energy harvesting, energy transfer, wireless sensor networks, interference channel, convex approximation, capacity assignment problem, Lagrange duality

## Abstract

In this work, we investigate the capacity allocation problem in the energy harvesting wireless sensor networks (WSNs) with interference channels. For the fixed topologies of data and energy, we formulate the optimization problem when the data flow remains constant on all data links and each sensor node harvests energy only once in a time slot. We focus on the optimal data rates, power allocations and energy transfers between sensor nodes in a time slot. Our goal is to minimize the total delay in the network under two scenarios, i.e., no energy transfer and energy transfer. Furthermore, since the optimization problem is non-convex and difficult to solve directly, by considering the network with the relatively high signal-to-interference-plus-noise ratio (SINR), the non-convex optimization problem can be transformed into a convex optimization problem by convex approximation. We attain the properties of the optimal solution by Lagrange duality and solve the convex optimization problem by the CVX solver. The experimental results demonstrate that the total delay of the energy harvesting WSNs with interference channels is more than that in the orthogonal channel; the total network delay increases with the increasing data flow for the fixed energy arrival rate; and the energy transfer can help to decrease the total delay.

## 1. Introduction

Energy harvesting is a promising solution to provide self-sustainability and extend the lifetime for energy-limit wireless sensor networks (WSNs) [1,2]. Thus, it has attracted much attention from researchers in recent years [3]. However, the energy harvesting process from the natural environment and the radio frequency signals [4] is instable, due to the time change of the day, the season or other factors [5]. Wireless energy transfer (WET) [6,7], as a friendly means of compensating energy, can transfer energy from some energy-rich sensor nodes to others with energy-hungry sensor nodes so as to enhance the overall network performance [8]. Meanwhile, due to the broadcast nature of wireless communications, the data signals of simultaneous transmissions cannot avoid interfering with each other in the same frequency band [9]. As a result, it decreases the network performance.

Because of these considerations, we investigate the energy harvesting WSNs and concentrate on the delay minimization problem of the WSNs with interference channels. The delay of every data link is determined by the information rate on the link, which is monotonically decreased as the rate of the link for the fixed data flow over it [10]. The information rate is monotonically increasing in SINR. We focus on the capacity assignment problem, which is similar to Bertsekas et al. [10]. In particular, compared with the special case, in which information and energy transfer channels are orthogonal to each other [11], we consider the general case of the communication model. In other words, the data transmission channels interfere with each other. This is a more realistic and meaningful model for the capacity assignment problem.

Therefore, by considering the energy consumption and power allocation for the fixed data flow, we formulate the capacity assignment problem in the energy harvesting WSNs with interference channels as a non-convex optimization problem. It is constrained by data flow conservation conditions, information rate requirements, energy and power consumption. Employing the relatively high SINR, the non-convex optimization problem can be transformed into a convex optimization problem by convex approximation in “log-sum-exp” form [12]. The solution properties of the transformed capacity allocation problem are derived by Lagrange duality. Then, it is available to search the optimal Lagrange multiplier and obtain the optimal solution to minimize total delay for the energy harvesting WSNs with interference channels in a time slot. Finally, we solve the approximate convex problem by the CVX solver [13].

Our study is related to and based on the previous classical works on a capacity allocation problem in communication networks [10]. In [14], the simultaneous routing and resource allocation (SRRA) are investigated. A capacitated multi-commodity flow model is used to describe the data flows in the wireless networks. The optimization problem is solved by the dual-decomposition method. A general flow-based analytical framework is presented in [15]. In order to balance aggregate user utility, total network cost, power control, rate allocation, routing and congestion control are jointly optimized in wireless networks. Channel-aware decision fusion by MIMO channels is investigated in an existing large antenna-array at the decision fusion center [16]. A decentralized multi-sensor estimation problem is studied in [17]. In a WSN with a coherent MAC, the detection and estimation of a zero-mean Gaussian signal is investigated in [18]. In [19], sensors simultaneously report sensed data to a fusion center with multiple antennas in a WSN and a Gaussian mixture channel model is adopted to attain a general fading description of the channels collective between the sensors and the fusion center. A machine learning based method is proposed for joint scheduling and power control in [20]. However, the previous works have not considered the energy harvesting and energy cooperation. Fouladgar et al. [21] investigates the optimization problem of simultaneous information and energy flows in graph-based communication networks with energy transfer. In [22], a model of multi-hop information transmission and energy transfer in TDMA-based multi-hop WSNs is proposed. Among previous studies, the most related to ours is that in [11], which investigates the delay minimization problem in the energy harvesting wireless communication networks with energy transfer. However, though Gurakan et al. [11], Fouladgar et al. [21] and Xu et al. [22] study the optimization problem of the joint information transmission and energy transfer, they neglect the interference among the data links. These motivate us to consider a general capacity assignment problem which is to minimize total delay in the energy harvesting WSNs with interference channels.

Lagrange method is a powerful studied tool which has been widely applied for the resource allocation problem in wireless networks [11,23]. It is worth noting that, although we utilize a similar mathematical approach to that in [11] for modeling and solving the capacity assignment problem, our study is significantly different from the previous studies: the previous studies only consider a special case where the data transmission channels are orthogonal to each other, rather than consider the impact of data transmission interference. However, the more realistic case is that data transmission channels interfere with each other, which is one of the critical issues to be tackled in this study. Therefore, we need to remodel the capacity assignment problem for the energy harvesting WSNs with interference channels in a time slot.

In this paper, our main contributions are as follows:

- We investigate a general and meaningful model of capacity assignment problem where the data links interfere with each other in the energy harvesting WSNs.
- Considering the relatively high SINR, we transform the non-convex optimization problem into a convex one by convex approximation, and also derive the optimal solution properties by Lagrange duality.
- Numerical results show that the interference signals significantly affect the network performance; the energy transfer can help to decrease the total network delay.

The rest of this paper is structured as follows. Section 2 introduces the network model and problem formulation. Section 3 investigates the capacity assignment problem with interference channels in a time slot. Section 4 demonstrates the performance results. Finally, Section 5 concludes the paper.

**Notations**. Throughout this paper, matrices and vectors are denoted by boldface uppercase and lowercase letters, respectively. log(·) stands for the natural logarithms. All numbers, vectors and matrices take real values in this paper. For a vector a, $a_{i}$ is the *i*th element; similarly, $a_{ij}$ denotes the (i,j)th entry of matrix A.

## 2. System Model and Problem Formulation

**Figure 1:**
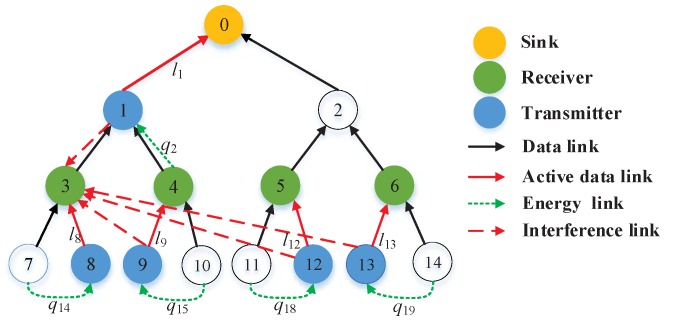
Interference channel model of data flows with the half-duplex mode.

In this study, each sensor node not only has the capability of harvesting energy and sensing data from the ambient environment, but it also can transmit or receive energy and data. As the data transmission channels interfere with each other, the interference signals among the data flow may be unavoidable. Hence, we consider an energy harvesting WSNs model with interference channels as shown in Figure 1.

Let G=(V,E) be a directed graph modeling *N* sensor nodes which are placed randomly and seamlessly in a certain area. The vertices set *V* = {$v_{0},v_{1},\ldots ,v_{N}$} is composed of one sink node and *N* sensor nodes. The edges set *E* is composed of the communication links between the sensor nodes, i.e., $(v_{i},v_{j})\in E$, if and only if a node $v_{i}$ can send a message to a node $v_{j}$ with the power constraint $p_{ij}$.

A data collection tree $T=(V_{T},E_{T})$ [24] is constructed for the energy harvesting WSNs with sink $v_{0}$ at level 0. It is an acyclic spanning subgraph of G=(V,E) where $V_{T}=V$ and $E_{T}\subseteq E$. In the data collection tree *T*, each sensor node $v_{n}$ can collect the sensing data from the area of interest and then store it for future transmission in a data buffer. Each sensor node $v_{n}$ has to send the sensing data to sink $v_{0}$ periodically in multi-hop fashion and half-duplex mode under the interference channel. Sensor nodes $v_{i}$ and $v_{j}$ are siblings if they have the same parent. Note that a sensor node can be either a transmitter, a relay or a receiver, which is determined by its location in WSNs. For brevity, the ordered pair $\left(v_{i},v_{j}\right)$ is replaced by (i,j) in the following sections (Throughout the paper, we denote sensor node indices by the first subscripts *i*, *j* and *n*. The subscript *i* and *j* denote the start node and the end node at each link (i.e., data link and energy link), respectively.).

We consider the following interference model to characterize the relationships among data links in tree-based energy harvesting WSNs. For any data link *l*, let $p_{l}\in \left(0,p_{l}^{max}\right]$ be the depleted power which transmits data flow from the sensor node $v_{i}$ to the sensor node $v_{j}$ in a time slot. We employ $p=\{p_{l}|l\in E_{T}\}$ as transmission power vector. Then, the received SINR of data link *l* is

(1) $$SINR_{l}\left(p\right)=\frac{G_{ll}p_{l}}{\sum_{\bar{l}\neq l}G_{\bar{l}l}p_{\bar{l}}+\sigma_{l}},$$

where $G_{\bar{l}l}$ is the channel gain from the transmitter of data link $\bar{l}$ to the receiver of data link *l*, which is dependent on various factors such as path loss, shadowing and fading effects. Particularly, $G_{ll}$ is the gain of primary link *l* and $\sigma_{l}$ denotes the channel noise power [25]. We adopt a simple distance based on the path-loss model to calculate the data link gains as $G_{ll}=d_{l}^{-\alpha }$, where α is constant between 3 and 4, which depends on the ambient conditions [26]. We assume that the channel gain remains constant and does not change over the time slot.

To illustrate, Figure 1 shows a tree-based energy harvesting WSNs with interference channels. In the figure, there are only five active links at the first time slot since we employ half-duplex sensor nodes [26]. Meanwhile, the network has five energy cooperation links, which can transfer energy to sensor nodes’ required energy. It guarantees that the sensing data can be successfully sent to the receivers at the time slot. In Figure 1, we assume that the active link $l_{8}$ is the primary link, the receiver $v_{3}$ not only receives the data flow signal from the transmitter $v_{8}$, but also receives the interference signals from other transmitters $v_{1}$, $v_{9}$, $v_{12}$ and $v_{13}$. The interference signals are represented by red dashed lines with arrows. Meanwhile, the sensor node $v_{7}$ can transfer energy to the sensor node $v_{8}$ through the energy link $q_{14}$. At the same time, other receivers also receive interference signals from active links’ transmitters except themselves. For brevity, we do not label them in Figure 1.

### 2.1. Network Data Flow Model

Let us denote the data link (i,j) as l∈1,…,L (The data link can be denoted (i,j) or *l*, they can be interchangeable in this paper). The topology of data flows can be described by an N×L matrix A. The entries of matrix A can be defined by $a_{nl}$, which is incident with sensor node *n* and data link *l*. More precisely, each entry $a_{nl}$ is defined as

(2) $$a_{nl}=\left\{\begin{array}{cc} 1, & \mathrm{if}\;n=i,\\ -1, & \mathrm{if}\;n=j,\\ 0, & \mathrm{otherwise}. \end{array}\right.$$

Let us define $I_{d}\left(n\right)$ as the set of incoming data links to sensor node $v_{n}$ and $O_{d}\left(n\right)$ as the set of outgoing data links from sensor node $v_{n}$, respectively. Assume that the data flow $d_{l}$ on each data link follows the uniform distribution U(0,a]. The set of data flows $\left\{d_{l}|l\in E_{T}\right\}$ is referred to as the *L*-dimensional flow vector. The divergence vector s associated with the data flow vector d is an *N*-dimensional vector which indicates the nonnegative amount of outside data flow injected into the sensor node $v_{n}$. Suppose that the data flow is lossless over links. For every sensor node $v_{n}$, the flow conservation conditions can be expressed as

(3) $$s_{n}=\sum_{l\in O_{d}\left(n\right)}d_{l}-\sum_{l\in I_{d}\left(n\right)}d_{l},\;\;\forall n.$$

The data flow conservation through the total WSNs can be rewritten as

(4) Ad=s.

Moreover, the data flow $d_{l}$ over each data link *l* cannot exceed the information carrying capacity $c_{l}$, i.e.,

(5) $$d_{l}\leq c_{l},\;\;\forall l.$$

### 2.2. Network Energy Flow Model

In this section, we present the energy model for the case where each sensor node has a single energy harvest in a time slot. Notice that we only consider harvested energy from the ambient environment and transferred energy from the neighbor sensor node, and ignore the energy contributed by interference in this paper.

#### 2.2.1. Energy Harvesting Model

Each sensor node powered can harvest energy from the ambient environment. Since the transmission consumption is the most significant amount of energy, we only account for energy consumption of transmitting data in this study. It is assumed that the energy harvesting sensor node has a capacity battery $B_{max}$ which is enough for transmitting the data. The capacity of storage is considered to be constant, i.e., energy outage and circuitry cost are negligible. Since energy harvesting sources have a random nature, the energy arrivals are considered as an independent and identically distributed (i.i.d.) Poisson distribution P(λ) with parameter λ [27,28]. We assume that the energy arrivals occur only once in a time slot. Let $E_{n}$ be the harvested energy of a sensor node $v_{n}$ in a time slot, $E_{n}\in \left(0,B_{max}\right]$. The harvested energy in a time slot can be exploited only in a later time slot.

#### 2.2.2. Energy Cooperation Model

Energy cooperation depends upon the statistics of the energy harvesting and the energy consumption of the sensor nodes. In general, for a sensor node $v_{n}$, the more data flow is transmitted, the more energy is required. In order to replenish the energy of energy-hungry sensor nodes, the technique of wireless energy cooperation [29] is adopted in our study. It is assumed that the energy is unidirectionally transferred from the sensor node $v_{i}$ to the sensor node $v_{j}$ in a time slot, the transfer efficiency is $\eta_{ij}$, $\eta_{ij}\in \left(0,1\right]$, due to energy loss in transmission and conversion.

#### 2.2.3. Energy Flow Model

In the previous analysis, we utilize *N*-dimensional vector E to present the harvested energy vector for the WSNs. In the energy transfer process, the wireless energy links are similar to data links. The wireless energy link *q* is also denoted as an ordered pair (i,j) in energy routing. The energy can be sent from the sensor node $v_{i}$ to the sensor node $v_{j}$ over energy link *q*, q∈1,…,Q, if the energy of the sensor node $v_{j}$ is not enough energy to operate. The energy transfer efficiency is $\eta_{q}$ on each energy link *q* where $\eta_{q}\in \left(0,1\right]$. It implies that $\delta_{i}$ amount of energy is transferred on wireless energy link *q* from the sensor node $v_{i}$ to the sensor node $v_{j}$, and the sensor node $v_{j}$ receives $\eta_{q}\delta_{i}$ amount of energy. The request for energy transfer is known in advance, whereas the amount of transferred energy is unknown. The topology of energy flow can be denoted by an N×Q matrix B. The entries of the matrix B can be defined by $b_{nq}$, which is incident with sensor node *n* and wireless energy link *q*. More specifically, each entry $b_{nq}$ can be described as [11]

(6) $$b_{nq}=\left\{\begin{array}{cc} 1, & \mathrm{if}\;n=i,\\ -\eta , & \mathrm{if}\;n=j,\\ 0, & \mathrm{otherwise}. \end{array}\right.$$

We define $O_{q}\left(n\right)$ and $I_{q}\left(n\right)$ as the set of outgoing and incoming wireless energy links at the sensor node $v_{n}$, respectively. The variable $x_{q}$ is the amount of energy transferred. Let vector x be the *L*-dimensional energy flow vector.

### 2.3. Communication Model

For the energy harvesting WSNs with interference channels, we focus on minimizing the total delay and enhancing the network performance in order to ensure that sensing data on each data link can reach the sink as quickly as possible. It is similar to [10,11]; we assume that each time slot is large enough and the delay on the data link *l* follows the M/M/1 queueing model in this work. It can be defined as

(7) $$D_{l}=\frac{d_{l}}{c_{l}-d_{l}},$$

where $d_{l}$ is the amount of data flow and $c_{l}$ is the information carrying capacity of communication link *l* in which $d_{l}\leq c_{l},\forall l\in E_{T}$.

According to the Shannon formula [11,30], the information carrying capacity (or information rate) $c_{l}$ of data link *l* can be expressed as

(8) $$c_{l}=\frac{1}{2}\log\left(1+SINR_{l}\left(p\right)\right),$$

where all logarithms in our study are taken to the base *e*.

At every sensor node $v_{n}$, the total power depleted (In contrast to transmission power consumption, the energy consumption of sensing data is ignored in our study.) on transmission data link *l* and energy link *q* are constrained by the usable energy as:

(9) $$\sum_{l\in O_{d}\left(n\right)}p_{l}\leq E_{n}+\sum_{q\in I_{q}\left(n\right)}\eta_{q}x_{q},\forall n.$$

Let $K=A^{+}$, where ${\left(a^{+}\right)}_{nl}=max\left\{a_{nl},0\right\}$, which only distinguish the outgoing links at each sensor node *n*. Hence, the energy availability constraints in Equation (9) can be rewritten as

(10) Kp+Bx≤E.

Notice that the power and energy can be interchangeable in a unit of time slot in this paper.

## 3. Capacity Assignment Problem in Energy Harvesting WSNs with Interference Channels

We consider the capacity assignment problem in WSNs with interference channels for a single energy harvesting sensor node in a time slot. Assume that the data flow assignments $d_{l}$ on all data links are fixed and available for harvested energy and transferred energy. The total delay *D* in a WSNs is

(11) $$D=\sum_{l\in E_{T}}\frac{d_{l}}{c_{l}-d_{l}}.$$

Hence, the goal of minimizing total delay in the energy harvesting WSNs with interference channels can be written as

(12a) $$\begin{array}{cc} \min_{c_{l},p_{l},x_{q}} & \sum_{l\in E_{T}}\frac{d_{l}}{c_{l}-d_{l}}, \end{array}$$

(12b) $$\begin{array}{cc} s.t.\; & \mathrm{Kp}+\mathrm{Bx}\leq E, \end{array}$$

(12c) $$\begin{array}{c} d_{l}\leq c_{l},\forall l, \end{array}$$

(12d) $$\begin{array}{c} x_{q}\geq 0. \end{array}$$

As shown in Figure 1, because the data transmission signals of active links interfere with each other, each data flow signal cannot perform interference cancelation and is treated as an additive noise compared with the primary link signal. By utilizing the information rate $c_{l}$ in Equation (8), the minimizing total delay in the energy harvesting WSNs with interference channels is

(13a) $$\begin{array}{cc} \min_{p_{l},x_{q}} & \sum_{l\in E_{T}}\frac{d_{l}}{\frac{1}{2}\log\left(1+\frac{G_{ll}p_{l}}{\sum_{\bar{l}\neq l}G_{\bar{l}l}p_{\bar{l}}+\sigma_{l}}\right)-d_{l}}, \end{array}$$

(13b) $$\begin{array}{cc} s.t.\; & \mathrm{Kp}+\mathrm{Bx}\leq E, \end{array}$$

(13c) $$\begin{array}{c} p_{l}\geq \frac{\sum_{\bar{l}\neq l}G_{\bar{l}l}p_{\bar{l}}+\sigma_{l}}{G_{ll}}\left(e^{2d_{l}}-1\right),\forall \;l, \end{array}$$

(13d) $$\begin{array}{c} x_{q}\geq 0. \end{array}$$

By analyzing Equation (13), we find that the minimizing of the total delay depends on the maximizing of the information carrying capacity $c_{l}$. Meanwhile, because the information carrying capacity $c_{l}$ is a monotonically increasing function of $SINR_{l}\left(p\right)$, the maximizing of information carrying capacity $c_{l}$ depends on the maximizing of the $SINR_{l}\left(p\right)$.

Note that the optimization problem (13) is non-convex since both the objective function (13a) and the constraint condition (13c) are non-convex in terms of transmission power vector p, and it is not straightforward to attain the optimal solution. Therefore, we need to study the fundamental properties of the optimization problem (13) and transform it into the convex optimization problem.

### 3.1. Convex Approximation

We can get a convex approximation for capacity assignment problem with interference channels when the SINRs are relatively high (e.g., SINRs ≥ 5 or 10). The information carrying capacity (or information rate) $c_{l}$ by using the Equation (1) can be rewritten as

(14) $$\begin{array}{cc} c_{l}\left(p\right) & \approx \frac{1}{2}\log\left(SINR_{l}\left(p\right)\right)\\ & =\frac{1}{2}\log\left(\frac{G_{ll}p_{l}}{\sum_{\bar{l}\neq l}G_{\bar{l}l}p_{\bar{l}}+\sigma_{l}}\right)\\ & =-\frac{1}{2}\log\left(\frac{\sum_{\bar{l}\neq l}G_{\bar{l}l}p_{\bar{l}}+\sigma_{l}}{G_{ll}p_{l}}\right)\\ & =-\frac{1}{2}\log\left(\frac{\sigma_{l}p_{l}^{-1}}{G_{ll}}+\frac{\sum_{\bar{l}\neq l}G_{\bar{l}l}p_{\bar{l}}p_{l}^{-1}}{G_{ll}}\right). \end{array}$$

Let ${\tilde{p}}_{l}=\log\left(p_{l}\right)$, i.e., $p_{l}=e^{{\tilde{p}}_{l}}$ for $l\in E_{T}$, we define

(15) $$\begin{array}{cc} \tilde{c_{l}}\left(\tilde{p}\right) & =c_{l}\left(p\left(\tilde{p}\right)\right)\\ & =-\frac{1}{2}\log\left(\frac{\sigma_{l}e^{-{\tilde{p}}_{l}}}{G_{ll}}+\frac{\sum_{\bar{l}\neq l}G_{\bar{l}l}e^{{\tilde{p}}_{\bar{l}}-{\tilde{p}}_{l}}}{G_{ll}}\right), \end{array}$$

where the functions $\tilde{c_{l}}\left(\tilde{p}\right)$ are concave in the vector $\tilde{p}$.

With the approximation information carrying capacity formula, the optimization problem (13) can be reformulated as

(16a) $$\begin{array}{c} \min_{{\tilde{p}}_{l},x_{q}}\sum_{l\in E_{T}}\frac{d_{l}}{-\frac{1}{2}\log\left(\frac{\sigma_{l}e^{-{\tilde{p}}_{l}}}{G_{ll}}+\frac{\sum_{\bar{l}\neq l}G_{\bar{l}l}e^{{\tilde{p}}_{\bar{l}}-{\tilde{p}}_{l}}}{G_{ll}}\right)-d_{l}}, \end{array}$$

(16b) $$\begin{array}{cc} s.t.\; & \mathrm{Kp}+\mathrm{Bx}\leq E, \end{array}$$

(16c) $$\begin{array}{c} e^{{\tilde{p}}_{l}}\geq \frac{\sum_{\bar{l}\neq l}G_{\bar{l}l}e^{{\tilde{p}}_{\bar{l}}}+\sigma_{l}}{G_{ll}}e^{2d_{l}},\forall \;l, \end{array}$$

(16d) $$\begin{array}{c} x_{q}\geq 0, \end{array}$$

where the objective function (16a) is a convex function in the new variable ${\tilde{p}}_{l}$ [12]. The information carrying capacity constraint (16c) is convex function in ${\tilde{p}}_{l}$ and $d_{l}$. This means that the optimization problem (16) is a convex optimization problem and the global optimal solution can be found.

**Remark** **1.** *Here, we use the approximation*$\frac{1}{2}\log\left(1+SINR_{l}\left(p\right)\right)\approx \frac{1}{2}\log\left(SINR_{l}\left(p\right)\right)$*which is reasonable for the optimization problem *(13)*, since*$\frac{1}{2}\log\left(SINR_{l}\left(p\right)\right)\leq \frac{1}{2}\log\left(1+SINR_{l}\left(p\right)\right)$*. This implies that the approximation is an underestimate and a tighter constraint for the information carrying capacity*$c_{l}\left(p\right)$. *Therefore, the solution of convex problem *(16)* is always feasible for the original optimization problem *(13)*.*

### 3.2. Properties of Capacity Assignment Problem with Interference Channels

For convex optimization problem (16), we form the dual problem by introducing Lagrange multiplier $\lambda \in R^{N}$, $\beta \in R^{L}$ and $\gamma \in R^{Q}$. The Lagrangian function is given by

(17) $$\begin{array}{c} L({\tilde{p}}_{l},x_{q},\lambda ,\beta ,\gamma )\\ =\sum_{l\in E_{T}}\frac{d_{l}}{-\frac{1}{2}\log\left(\frac{\sigma_{l}e^{-{\tilde{p}}_{l}}}{G_{ll}}+\frac{\sum_{\bar{l}\neq l}G_{\bar{l}l}e^{{\tilde{p}}_{\bar{l}}-{\tilde{p}}_{l}}}{G_{ll}}\right)-d_{l}}\\ +\sum_{n}\lambda_{n}\left(\sum_{l\in O_{d}\left(n\right)}e^{{\tilde{p}}_{l}}-E_{n}-\sum_{q\in I_{q}\left(n\right)}\eta_{q}x_{q}\right)\\ -\sum_{l\in E_{T}}\beta_{l}\left(e^{{\tilde{p}}_{l}}-\frac{\sum_{\bar{l}\neq l}G_{\bar{l}l}e^{{\tilde{p}}_{\bar{l}}}+\sigma_{l}}{G_{ll}}e^{2d_{l}}\right)-\sum_{q}\gamma_{q}x_{q}. \end{array}$$

The Lagrangian function (17) corresponds to Lagrange dual function $\bar{Q}:R^{N}\times R^{L}\times R^{Q}\to R$ as

(18) $$\begin{array}{cc} \bar{Q}\left(\lambda ,\beta ,\gamma \right) & =\underset{{\tilde{p}}_{l},x_{q}}{\inf}L\left({\tilde{p}}_{l},x_{q},\lambda ,\beta ,\gamma \right). \end{array}$$

The dual optimization problem is

(19a) $$\begin{array}{c} \max\;\bar{Q}\left(\lambda ,\beta ,\gamma \right), \end{array}$$

(19b) $$\begin{array}{c} s.t.\;\lambda \geq 0,\beta \geq 0,\gamma \geq 0. \end{array}$$

The KKT optimality conditions hold for the convex optimization problem (16), thus we have

(20) $$\begin{array}{c} \frac{\partial L}{\partial {\tilde{p}}_{l}}=\frac{\partial t_{l}\left({\tilde{p}}_{l}\right)}{\partial {\tilde{p}}_{l}}+e^{{\tilde{p}}_{l}}\left[\lambda_{i(l)}-\left(\beta_{l}-\beta_{\bar{l}}\sum_{\bar{l}\neq l}\frac{G_{l\bar{l}}e^{2d_{\bar{l}}}}{G_{\bar{l}\bar{l}}}\right)\right]=0,\forall l,\bar{l} \end{array}$$

(21) $$\frac{\partial L}{\partial x_{q}}=-\eta_{q}\lambda_{j(q)}-\gamma_{q}=0,\forall i,j\in V_{T},\;\forall q,$$

where

(22) $$\begin{array}{c} t_{l}\left({\tilde{p}}_{l}\right)≜d_{l}{\left[-\frac{1}{2}\log\left(\frac{\sigma_{l}e^{-{\tilde{p}}_{l}}}{G_{ll}}+\frac{\sum_{\bar{l}\neq l}G_{\bar{l}l}e^{{\tilde{p}}_{\bar{l}}-{\tilde{p}}_{l}}}{G_{ll}}\right)-d_{l}\right]}^{-1}. \end{array}$$

The complementary slackness conditions are

(23) $$\begin{array}{c} \lambda_{n}\left(\sum_{l\in O_{d}\left(n\right)}e^{{\tilde{p}}_{l}}-E_{n}-\sum_{q\in I_{q}\left(n\right)}\eta_{q}x_{q}\right)=0,\forall n, \end{array}$$

(24) $$\beta_{l}\left(e^{{\tilde{p}}_{l}}-\frac{\sum_{\bar{l}\neq l}G_{\bar{l}l}e^{{\tilde{p}}_{\bar{l}}}+\sigma_{l}}{G_{ll}}e^{2d_{l}}\right)=0,\forall l,$$

(25) $$\gamma_{q}x_{q}=0,\;\forall q.$$

We extend Lemmas 1 and 2 in [11] and derive some properties about the optimal power allocation with interference channels as follows.

**Lemma** **1.** *The feasibility of the convex optimization problem *(16)* requires*$\beta_{l}=0,\forall l$.

**Proof.**  The proof is a similar procedure in [11]. If the convex optimization problem (16) is feasible, the objective function (16a) must be bounded. The constraint condition (16c) for any data link *l* means that the objective function (16a) is unbounded. Thus, the constraint condition (16c) must strictly satisfy the inequalities for all data link *l*. From Equation (24), we can conclude that $\beta_{l}=0,\forall l$. □

**Lemma** **2.** *At each sensor node* $v_{n}$ *, the optimal power allocation with interference channels among data links satisfies*

(26) $$\frac{\partial t_{l}\left({\tilde{p}}_{l}\right)}{\partial {\tilde{p}}_{l}}=\frac{\partial t_{i}\left({\tilde{p}}_{i}\right)}{\partial {\tilde{p}}_{i}},\forall l,\forall i\in O_{d}\left(n\right).$$

**Proof.**  The proof is a similar procedure in [11]. Combining Equation (20) and Lemma 1, we attain

(27) $$\frac{\partial t_{l}\left({\tilde{p}}_{l}\right)}{\partial {\tilde{p}}_{l}}=-e^{{\tilde{p}}_{l}}\lambda_{i(l)},\forall l.$$

Since the outgoing links *l* and *i* reside in the same sensor node *n*, we have

(28) $$\frac{\partial t_{l}\left({\tilde{p}}_{l}\right)}{\partial {\tilde{p}}_{l}}=-e^{{\tilde{p}}_{l}}\lambda_{i}=\frac{\partial t_{i}\left({\tilde{p}}_{i}\right)}{\partial {\tilde{p}}_{i}}.$$

Thus, we can conclude that Equation (26) holds. □

In the next subsections, we separately solve the convex optimization problem (16) under two cases, i.e., no energy transfer and energy transfer.

### 3.3. Case without Energy Transfer

As energy transfer does not occur in this case, we have $x_{q}=0,\forall q$. Thus, the convex optimization problem (16) becomes only in respect of ${\tilde{p}}_{l}$ as follows:

(29a) $$\begin{array}{cc} \min_{{\tilde{p}}_{l}} & \sum_{l\in E_{T}}\frac{d_{l}}{-\frac{1}{2}\log\left(\frac{\sigma_{l}e^{-{\tilde{p}}_{l}}}{G_{ll}}+\frac{\sum_{\bar{l}\neq l}G_{\bar{l}l}e^{{\tilde{p}}_{\bar{l}}-{\tilde{p}}_{l}}}{G_{ll}}\right)-d_{l}}, \end{array}$$

(29b) $$\begin{array}{cc} s.t.\; & \sum_{l\in O_{d}\left(n\right)}e^{{\tilde{p}}_{l}}\leq E_{n},\forall n\in V_{T}, \end{array}$$

(29c) $$\begin{array}{c} e^{{\tilde{p}}_{l}}\geq \frac{\sum_{\bar{l}\neq l}G_{\bar{l}l}e^{{\tilde{p}}_{\bar{l}}}+\sigma_{l}}{G_{ll}}e^{2d_{l}},\forall \;l. \end{array}$$

Since we employ *half-duplex* WSNs, the optimization problem can be considered L active data links in the energy harvesting WSNs with interference channels as

(30a) $$\begin{array}{cc} \min_{{\tilde{p}}_{l}} & \sum_{i=1}^{\bar{L}}\sum_{l\in O_{d}\left(n\right)}\frac{-2d_{l}}{\log\left(\frac{\sigma_{l}e^{-{\tilde{p}}_{l}}+\sum_{\bar{l}\neq l}G_{\bar{l}l}e^{{\tilde{p}}_{\bar{l}}-{\tilde{p}}_{l}}}{G_{ll}}\right)-d_{l}}, \end{array}$$

(30b) $$\begin{array}{cc} s.t.\; & \sum_{l\in O_{d}\left(n\right)}e^{{\tilde{p}}_{l}}\leq E_{n},\forall n\in V_{T}, \end{array}$$

(30c) $$\begin{array}{c} e^{{\tilde{p}}_{l}}\geq \frac{\sum_{\bar{l}\neq l}G_{\bar{l}l}e^{{\tilde{p}}_{\bar{l}}}+\sigma_{l}}{G_{ll}}e^{2d_{l}},\forall \;l. \end{array}$$

If the optimization problem (30) is feasible, then it requires

(31) $$\sum_{l\in O_{d}\left(n\right)}\frac{\sum_{\bar{l}\neq l}G_{\bar{l}l}e^{{\tilde{p}}_{\bar{l}}}+\sigma_{l}}{G_{ll}}e^{2d_{l}}\leq E_{n},$$

which we assume that it holds. Similar to Equations (17) and (30) corresponding to Lagrangian function $\hat{L}$ with $\lambda \in R^{N}$ is

(32) $$\begin{array}{c} \hat{L}\left({\tilde{p}}_{l},\lambda \right)\\ =\sum_{i=1}^{\bar{L}}\sum_{l\in O_{d}\left(n\right)}\frac{-2d_{l}}{\log\left(\frac{\sigma_{l}e^{-{\tilde{p}}_{l}}+\sum_{\bar{l}\neq l}G_{\bar{l}l}e^{{\tilde{p}}_{\bar{l}}-{\tilde{p}}_{l}}}{G_{ll}}\right)-d_{l}}+\sum_{n}\lambda_{n}\left(\sum_{l\in O_{d}\left(n\right)}e^{{\tilde{p}}_{l}}-E_{n}\right). \end{array}$$

Meanwhile, the KKT optimality condition is

(33) $$\frac{\partial \hat{L}}{\partial {\tilde{p}}_{l}}=\frac{\partial t_{l}\left({\tilde{p}}_{l}\right)}{\partial {\tilde{p}}_{l}}+e^{{\tilde{p}}_{l}}\lambda =0,\forall l\in O_{d}\left(n\right)$$

and the complementary slackness condition is

(34) $$\lambda \left(\sum_{l\in O_{d}\left(n\right)}e^{{\tilde{p}}_{l}}-E_{n}\right)=0,\;\forall l,$$

(35) $$\begin{array}{c} \frac{\partial t_{l}\left({\tilde{p}}_{l}\right)}{\partial {\tilde{p}}_{l}}\\ =-\frac{1}{2}d_{l}{\left[-\frac{1}{2}\log\left(\frac{\sigma_{l}e^{-{\tilde{p}}_{l}}}{G_{ll}}+\frac{\sum_{\bar{l}\neq l}G_{\bar{l}l}e^{{\tilde{p}}_{\bar{l}}-{\tilde{p}}_{l}}}{G_{ll}}\right)-d_{l}\right]}^{-2}\\ +\frac{1}{2}\sum_{\bar{l}\neq l}\left\{d_{\bar{l}}\left[-\frac{1}{2}\log\left(\frac{\sigma_{\bar{l}}e^{-{\tilde{p}}_{\bar{l}}}+\sum_{k\neq \bar{l}}G_{k\bar{l}}e^{{\tilde{p}}_{k}-{\tilde{p}}_{\bar{l}}}}{G_{\bar{l}\bar{l}}}\right)\right.\right.\\ \left.{\left.-d_{\bar{l}}\right]}^{-2}\left(\frac{G_{l\bar{l}}e^{{\tilde{p}}_{l}}}{\sigma_{\bar{l}}+\sum_{k\neq \bar{l}}G_{k\bar{l}}e^{{\tilde{p}}_{k}}}\right)\right\},\forall l,\bar{l},k. \end{array}$$

From Equation (33), we have

(36) $$\begin{array}{ll} \lambda & =-\frac{\partial t_{l}\left({\tilde{p}}_{l}\right)}{\partial {\tilde{p}}_{l}}e^{-{\tilde{p}}_{l}}\\ & =\frac{d_{l}}{2e^{{\tilde{p}}_{l}}}{\left[-\frac{1}{2}\log\left(\frac{\sigma_{l}e^{-{\tilde{p}}_{l}}}{G_{ll}}+\frac{\sum_{\bar{l}\neq l}G_{\bar{l}l}e^{{\tilde{p}}_{\bar{l}}-{\tilde{p}}_{l}}}{G_{ll}}\right)-d_{l}\right]}^{-2}\\ & -\sum_{\bar{l}\neq l}\left\{\frac{d_{\bar{l}}}{2}\left[-\frac{1}{2}\log\left(\frac{\sigma_{\bar{l}}e^{-{\tilde{p}}_{\bar{l}}}+\sum_{k\neq \bar{l}}G_{k\bar{l}}e^{{\tilde{p}}_{k}-{\tilde{p}}_{\bar{l}}}}{G_{\bar{l}\bar{l}}}\right)\right.\right.\\ & \left.{\left.-d_{\bar{l}}\right]}^{-2}\left(\frac{G_{l\bar{l}}}{\sigma_{\bar{l}}+\sum_{k\neq \bar{l}}G_{k\bar{l}}e^{{\tilde{p}}_{k}}}\right)\right\},\forall l,\bar{l},k. \end{array}$$

For the total energy constraint condition Equation (30b), the optimal power allocation can be found by searching the optimal $\lambda^{*}$.

**Remark** **2.** *The constraint condition *(30c)* is not included in the Lagrangian function *(32)*, since the constraint condition *(30c)* will always hold when the convex optimization problem *(30)* is feasible.*

### 3.4. Case with Energy Transfer

Next, we solve the case with energy transfer, which implies $x_{q}\geq 0$ for some energy links *q*. The convex optimization problem (16) becomes

(37a) $$\begin{array}{cc} \min_{{\tilde{p}}_{l},x_{q}} & \sum_{l\in E_{T}}\frac{d_{l}}{-\frac{1}{2}\log\left(\frac{\sigma_{l}e^{-{\tilde{p}}_{l}}}{G_{ll}}+\frac{\sum_{\bar{l}\neq l}G_{\bar{l}l}e^{{\tilde{p}}_{\bar{l}}-{\tilde{p}}_{l}}}{G_{ll}}\right)-d_{l}}, \end{array}$$

(37b) $$\begin{array}{cc} s.t.\; & \sum_{l\in O_{d}\left(n\right)}e^{{\tilde{p}}_{l}}\leq E_{n}+\sum_{q\in I_{q}\left(n\right)}\eta_{q}x_{q},\forall n, \end{array}$$

(37c) $$\begin{array}{c} e^{{\tilde{p}}_{l}}\geq \frac{\sum_{\bar{l}\neq l}G_{\bar{l}l}e^{{\tilde{p}}_{\bar{l}}}+\sigma_{l}}{G_{ll}}e^{2d_{l}},\forall \;l, \end{array}$$

(37d) $$\begin{array}{c} x_{q}\geq 0. \end{array}$$

According to the *half-duplex* mode, the optimization problem (37) which has $\bar{L}$ active data links in the energy harvesting WSNs with interference channels can be written as

(38a) $$\begin{array}{cc} \min_{{\tilde{p}}_{l},x_{q}} & \sum_{i=1}^{\bar{L}}\sum_{l\in O_{d}\left(n\right)}\frac{d_{l}}{-\frac{1}{2}\log\left(\frac{\sigma_{l}e^{-{\tilde{p}}_{l}}}{G_{ll}}+\frac{\sum_{\bar{l}\neq l}G_{\bar{l}l}e^{{\tilde{p}}_{\bar{l}}-{\tilde{p}}_{l}}}{G_{ll}}\right)-d_{l}}, \end{array}$$

(38b) $$\begin{array}{cc} s.t.\; & \sum_{l\in O_{d}\left(n\right)}e^{{\tilde{p}}_{l}}\leq E_{n}+\sum_{q\in I_{q}\left(n\right)}\eta_{q}x_{q},\forall n, \end{array}$$

(38c) $$\begin{array}{c} e^{{\tilde{p}}_{l}}\geq \frac{\sum_{\bar{l}\neq l}G_{\bar{l}l}e^{{\tilde{p}}_{\bar{l}}}+\sigma_{l}}{G_{ll}}e^{2d_{l}},\forall \;l, \end{array}$$

(38d) $$\begin{array}{c} x_{q}\geq 0. \end{array}$$

As in Section 2.2.2, it is assumed that some energy $x_{q}>0$ is transferred from the sensor node $v_{i}$ to the sensor node $v_{j}$ over energy link *q*. Since sensor node $v_{i}$ only transfers energy and does not transmit data, the energy causality constraint condition on sensor node $v_{j}$ is denoted as

(39) $$\sum_{l\in O_{d}\left(j\right)}e^{{\tilde{p}}_{l}}\left(\lambda_{j}^{*}\right)=E_{j}+\eta_{q}x_{q}.$$

Therefore, by combining Equations (36) and (39), we can attain optimal power allocations if we find the optimal $\lambda_{j}^{*}$.

The Lagrangian method can provide some ideas and in-depth insight into the above-defined optimization problem. However, it is difficult to find a closed-form optimal solution. Therefore, we use the CVX solver [13] to tackle the optimization problems (30) and (38) in this paper.

## 4. Simulation Results and Analysis

We provide some experimental results to demonstrate the resulting optimal energy-delay policies in the energy harvesting WSNs with interference channels. Note that we only consider the total delay of all active links in the network in a time slot, thus the power and energy can be interchangeable. We conduct our experiment on a PC with the Intel(R) Core (TM) i7-7700, 3.60 GHz CPU, 8 GB RAM and Windows 8 (version 6.2). We use CVX 2.1 [13] which is implemented in MATLAB 9.2 (version R2017a) to solve the optimization problems.

### 4.1. Simulation Results

**Figure 2:**
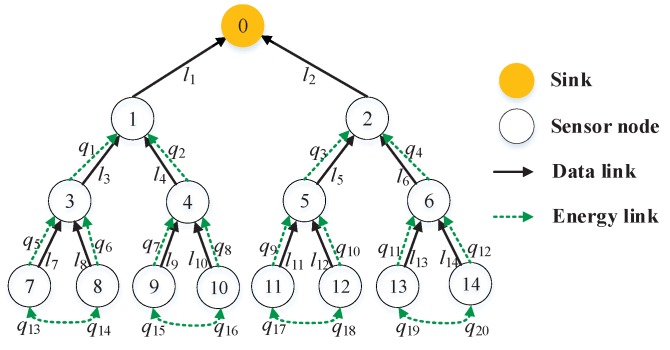
Data and energy topologies.

In the simulations, tree-based WSNs topologies are considered. Figure 2 shows the data and energy topologies in energy harvesting WSNs, which has one sink (i.e., $v_{0}$), 14 sensor nodes, 14 directed data links and 20 directed energy links. It is noted that each leaf sensor node only needs to transfer energy from its sibling neighboring sensor node; each parent sensor node needs to transfer energy from children sensor nodes in order to transmit successfully heavy sensing data from itself and children sensor nodes; and the sink node does not need to transfer energy since it is not energy-limited. Meanwhile, the half-duplex mode is adopted in the network system. In other words, there are only a few active links in a time slot. In Figure 1, we observe that there are five active links keeping simultaneous communication in the first time slot.

Each time slot, the energy arrivals follow an i.i.d Poisson distribution P(λ) with λ=8, and the data flow on each data link follows the uniform distribution U(0,a],a∈[0.5,1.5]. For ease of calculation, similar to Johansson et al. [31], all the receivers have the same noise power $\sigma_{l}=1\times 10^{-5}$ units; all diagonal entries of the channel gain matrix G are set to 1 and the off-diagonal entries are attained by the uniform distribution U(0,0.01]. Energy transfer efficiency $\eta_{q}$ is set to 0.6 on all energy links [32].

As an example, we adopt the data and energy topologies in Figure 1 to perform evaluation the optimization problem. The fixed data flows are $d={\left[d_{l_{1}},d_{l_{8}},d_{l_{9}},d_{l_{12}},d_{l_{13}}\right]}^{T}={\left[0.4585,0.8752,0.6869,0.2313,0.4887\right]}^{T}$ units. The energy arrival vector $E_{1}={\left[9,10,7,8,9\right]}^{T}$ units and $E_{2}={\left[11,10,8,4,6\right]}^{T}$ units denote transmitters $\left\{v_{1},v_{8},v_{9},v_{12},v_{13}\right\}$ and transferring energy sensor nodes $\left\{v_{4},v_{7},v_{10},v_{11},v_{14}\right\}$, respectively. The energy transfer efficiency vector is η = ${\left[0.6,0.6,0.6,0.6,0.6\right]}^{T}$ (Here, we only give data flow of active links, corresponding to the energy of sensor nodes and the efficiency of energy transfer. Notice that all variables are uniform units in this paper). The solution results of optimization problem under two scenarios (i.e., no energy transfer and energy transfer) are shown in the right half of Table 1. In order to further confirm the significance of our study, we also perform the optimization problem of the orthogonal channel [11] in the tree-based network topologies. The solution results are shown in the left half of Table 1.

**Figure 3:**
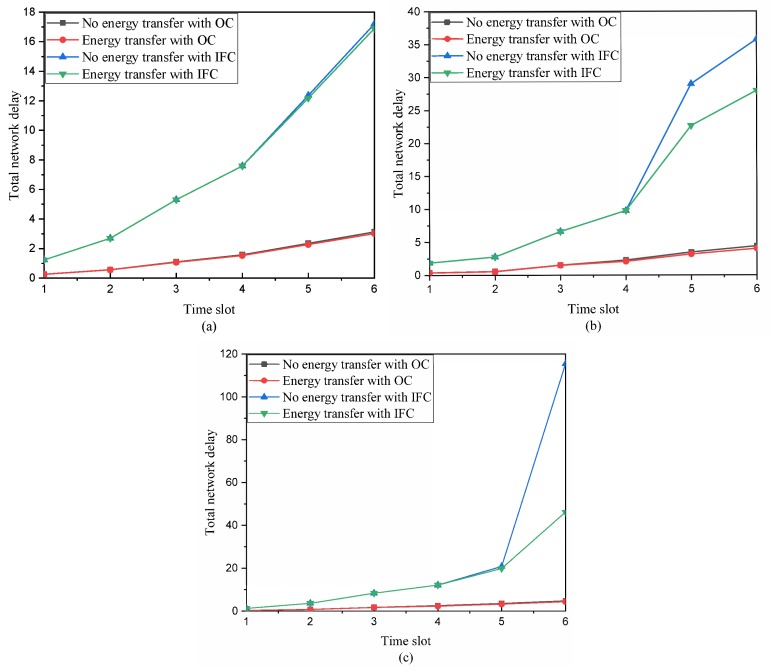
The total delay of energy harvesting WSNs over time: (**a**) Data flow d∼U(0,0.5]; (**b**) data flow d∼U(0,1]; (**c**) data flow d∼U(0,1.5].

To better evaluate the optimization problem, we consider the total delay of a data collection round [24] in the energy harvesting WSNs. A data collection round is a process where the sink collects sensing data from all sensor nodes; the sensing data is in turn transferred from leaf sensor nodes to sink over parent sensor nodes. In particular, the parent sensor nodes not only transmit received sensing data of child sensor nodes, but also transmit their own sensing data to their parent sensor nodes. In Figure 2, a data collection round is divided into six time slots according to the half-duplex communication mode. Using the same parameter settings, we perform the optimization problem under both orthogonal channel (OC) and interference channel (IFC) with no energy transfer and energy transfer, respectively. For three different data flow vectors, i.e., d∼U(0,0.5], d∼U(0,1] and d∼U(0,1.5], we attain the total network delay over time as shown in Figure 3.

### 4.2. Performance Analysis

From Table 1 and Figure 3, we observe that the network delay in the orthogonal channel is less than that in the interference channel. It means that the interference signals among data links significantly affect the total network delay in energy harvesting WSNs, which should not be ignored in the WSN design. Meanwhile, it can be seen that the total network delay increases with the increasing data flow for the fixed energy arrival rate in Figure 3.

From Figure 3, we also noticed that, during the earlier time slots, i.e., from time slot 1 to time slot 4, the performance difference of the network delay under both no energy transfer and energy transfer is insignificant in the two scenarios, i.e., orthogonal channel (OC) and interference channel (IFC). This is because there is enough energy at each sensor node to send the small amount of sensing data on each active data link. However, when the amount of sensing data which comes from their own and their descendant increases over time, the data links load increases, resulting in a lower contribution of the network delay. Meanwhile, when the energy arrival rate cannot increase over time, the sensing data remains more often in the buffer because there is not enough energy to transmit it. As a result, there is a relatively high network delay. This is particularly evident for the scenario with interference channels.

In the scenarios of orthogonal channel and interference channel, the network delay with no energy transfer is more than that with energy transfer especially during the later time slots, i.e., from time slot 5 to time slot 6 in Figure 3, since energy transfer between the energy-rich sensor nodes and the energy-hungry sensor nodes can help to decrease the total delay and enhance the total performance in WSNs.

We also noticed that the closer the sensor node is to the sink, the more energy is needed since it has heavier traffic loads. Meanwhile, the total network delay also increases for the fixed channel gain. Moreover, it can be seen that the power allocation of each active link is proportional to SINR in Table 1.

## 5. Discussion

Our work can be further extended in some aspects. First, the approximate method only suits for the case of the relatively high SINR and cannot be used to deal with the case of low SINR in the network. Second, we cannot provide a closed-form solution for the optimization problem and only employed the experimental results to explain the optimization problem, making it difficult to carry out theoretical analysis on the relationship between data flow and energy flow under interference channel in a time slot. Moreover, the network topology can be replaced by the others in our model and the distributed approach [33] can be considered in the large-scale energy harvesting WSNs. In the future, we will consider the above aspects and plan to show the proposed model on the real sensor network testbed. In addition, the joint optimization of capacity and flow under interference channel will be investigated, and the non-orthogonal multiple access technique [34], which is to support a great number of users, can be explored in energy harvesting WSNs for future works.

## 6. Conclusions

We have investigated the optimal data rates, power allocations and energy transfers for minimizing the total delay in the energy harvesting WSNs with interference channels in a time slot. We have formulated the optimization problem which is subject to information rate requirements, energy and power consumption as a non-convex optimization problem under two cases, i.e., no energy transfer and energy transfer. By exploiting the convex approximation with the relatively high SINR, the optimization problem has been converted into a tractable convex problem. Moreover, we also have derived the properties of the optimal solution by Lagrange duality. Finally, we solved the optimization problem by the CVX solver. The experimental results showed that, when data flow and energy topologies were fixed, the interference signals significantly affect the network performance; the total network delay increases with the increasing data flow for the fixed energy arrival rate; the energy transfer can help to decrease the total network delay; and the power allocation on each data link was proportional to SINR for the energy harvesting WSNs in a time slot. Moreover, we also have discussed the extension of our work.

## Figures and Tables

**Table 1:** Solution results of optimization problem under both orthogonal channel and interference channel in the first time slot.

Link	Orthogonal Channel					Interference Channel						
	No Energy Transfer		Energy Transfer			No Energy Transfer			Energy Transfer			
	Power	Delay	Power	TE	Delay	Power	SINR	Delay	Power	TE	SINR	Delay
$l_{1}$	8.8143		15.6000	11.0000		5.1660	78.6533		8.2649	7.9520	78.6532	
$l_{8}$	10.0000		16.0000	10.0000		10.0000	143.1230		16.0000	10.0000	143.1436	
$l_{9}$	7.0000	0.3740	11.8000	8.0000	0.3622	4.6663	57.5294	1.8858	7.4654	6.2319	57.5311	1.8857
$l_{12}$	6.4475		10.4000	4.0000		2.5360	14.3840		4.0573	1.2875	14.3839	
$l_{13}$	9.0000		12.6000	6.0000		3.5185	43.8209		5.6291	3.0528	43.8212	

Transferred energy is abbreviated as TE.
